# The expression of metalloproteinases in the lumbar disc correlates strongly with Pfirrmann MRI grades in lumbar spinal fusion patients

**DOI:** 10.1016/j.bas.2022.100872

**Published:** 2022-02-05

**Authors:** Sanjay S. Aripaka, Rachid Bech-Azeddine, Louise M. Jørgensen, Jens D. Mikkelsen

**Affiliations:** a Neurobiology Research Unit, University Hospital Copenhagen, Rigshospitalet, Copenhagen, Denmark; b Copenhagen Spine Research Unit, Center for Rheumatology and Spine Diseases, Rigshospitalet, Glostrup, Denmark; c Department of Clinical Medicine, Faculty of Health and Medical Sciences, University of Copenhagen, Copenhagen, Denmark; d Department of Neuroscience, Faculty of Health and Medical Sciences, University of Copenhagen, Copenhagen, Denmark

**Keywords:** ADAMTS, Disc Degeneration, MMP, mRNA, Real-time PCR, DDD, degenerative disc disease, MMP, matrix metalloproteinase, ADAMT, a disintegrin and metalloproteinases with thrombospondin motif, LBP, low back pain, NP, nucleus pulposus, AF, annulus fibrosus, ECM, extracellular matrix, IVD, intervertebral disc, MRI, magnetic resonance imaging

## Abstract

**Introduction:**

Increased catabolism of the extracellular matrix is observed under degenerative disc disease (DDD). The cleavage of extracellular matrix proteins in the nucleus pulposus (NP) by either matrix metalloproteinases (MMPs) or a disintegrin and metalloproteinases with thrombospondin motifs (ADAMTSs) is believed to be involved in the degeneration, but the mechanisms are not known.

**Research question:**

Here, we examine the correlation between expression of several MMPs and ADAMTSs subtypes in lumbar discs from 34 patients with low back pain (LBP) undergoing 1-2 level lumbar fusion surgery (L4/L5 and/or L5/S1) for DDD with or without spondylolisthesis.

**Materials and Methods:**

The mRNA levels of MMPs (subtypes 1, 2, 3, 10, and 13) and ADAMTSs (subtypes 1, 4, and 5) were analyzed using quantitative real-time polymerase chain reaction (RT-qPCR) and correlated to the Pfirrmann magnetic resonance imaging classification system (grade I-V) of lumbar DDD.

**Results:**

We find a highly significant positive correlation between Pfirrmann grades and the gene expression of MMP1 (r=0.67, p=0.0001), MMP3 (r=0.61, p=0.0002), MMP10 (r=0.6701, p=0.0001), MMP13 (r=0.48, p=0.004), ADAMTS1 (r=0.67, p=0.0001), and ADAMTS5 (r=0.53, p=0.0017). The similar regulation of these transcript suggests their involvement in disc degeneration. Interestingly, a post hoc analysis (uncorrected p-values) also demonstrated a positive correlation between expression of TNF-α, IL-6 and both ADAMTSs/MMPs and the Pfirrmann grades.

**Discussion and Conclusion:**

These findings show that disc degradation in DDD is strongly associated with the expression of some metalloproteinases.

## Highlights

• An imbalance between catabolism and anabolism of IVD matrix components.
• MMPs and ADAMTSs are expressed in the NP, and their expression levels increase with degeneration grade.
• Our results suggest that inflammatory cytokines participate in the regulation of MMPs and ADAMTSs.

## 1 Introduction

Low back pain (LBP) represents the top medical expense in western societies and is a leading cause of disability worldwide (Vos et al., 2016). LBP is age-related, and several lines of evidence show that the LBP is associated with degenerative disc disease (DDD) (Hadjipavlou et al., 2008) even the etiology of DDD is not fully understood. DDD is histologically characterized by cell number reduction, extracellular matrix (ECM) loss and inflammation (Risbud and Shapiro, 2014; Weber et al., 2015). The intervertebral disc (IVD) is an essential component of the spine and consists of an outer fibrous ring, the annulus fibrosus (AF), rich in collagen type I providing strength (Freemont, 2009) and the centrally located nucleus pulposus (NP), which is rich in collagen type II and proteoglycans, mainly aggrecan. In healthy discs, a careful regulation between growth factors and catabolic enzymes entails that the rate of synthesis and breakdown of the ECM is in equilibrium (Wang et al., 2014, 2015). Therefore, any loss of collagen and proteoglycans is key in developing DDD (Sivan et al., 2014). Matrix metalloproteinases (MMPs) and a disintegrin and metalloproteases with thrombospondin motifs (ADAMTSs) are families of enzymes that cleave collagens and proteoglycans, respectively, and we consider these molecules to be important in tissue degeneration in LBP and DDD.

MMPs are a large family of zinc-dependent proteolytic enzymes associated with extracellular matrix protein turnover and tissue degradation (Sivan et al., 2014; Basaran et al., 2014; Cawston et al., 2001; Nagase et al., 2006; Richardson et al., 2009). Currently, 24 MMP human subtypes have been identified and classified into six groups based on their substrate specificity (Haro et al., 1999). MMPs are usually secreted in inactive forms and require a regulatory activator protein to be active (Visse and Nagase, 2003). High expression of MMP-1,-3 is seen in recurrent disc herniation (Sivan et al., 2014), but whether expression of any MMP subtypes relates to the progression of the DDD is unknown.

The other major component of the NP along with collagens is proteoglycans, mainly aggrecan. Aggrecan is the most common proteoglycan that makes 50% of the dry weight of NP, which plays an important role in water absorbing capacity and also contributes in the diffusion of nutrients from periphery and in the maintenance of disc height and ability to withstand compressions (Wang et al., 2014; Adams and Roughley, 2006). Specific change associated with disc degeneration is the loss of proteoglycans which decreases the disc's water holding capacity (Oegema, 1993; Lyons et al., 1981). ADAMTSs are synthesized as pre-pro-enzymes later cleaved by furin or furin-like proteases to activated forms which are secreted and associated with proteolytic events of ECM components and leading to degradation (Dubail and Apte, 2015). Even though MMPs lyses aggrecan and collagens (Fosang et al., 1996a, 1996b), there is increasing evidence for the role of ADAMTSs in the degradation of aggrecan as well (Little et al., 1999; Nagase and Kashiwagi, 2003). Studies focused on osteoarthritis with disc degeneration showed upregulation in expression of ADAMTS in degenerated articular cartilage (Flannery et al., 1999; Vankemmelbeke et al., 2001). ADAMTS4 and ADAMTS5 have received increased attention because they can specifically cleave cartilage proteoglycans. Loss of aggrecan occurs during IVD tissue degeneration (Vo et al., 2013; Gendron et al., 2007). ADAMTS subtype 1, 4, 5, degrade aggrecan and the expression of the same subtypes is found to be increased in degenerated IVD tissue. Previous studies have reported the expression of MMPs and ADAMTSs in IVD tissues and their implication in catabolism and loss of ECM (Vo et al., 2013).

In our previous study, we showed increased expression of inflammatory cytokines correlates with pain intensity and disability in lumbar discs from patients undergoing lumbar spine fusion (Aripaka et al., 2021). The present study was aimed to gain better insight into the role of different MMP and ADAMTS expression in IVD’s from same patients and also study the relevance to inflammation, We therefore determined levels of MMP transcript subtypes (1, 2, 3, 10, and 13) and ADAMTS subtypes s (1, 4, and 5) as previously described (Sivan et al., 2014) in the NP and their association to lumbar disc degeneration in patients with chronic lumbar pain, as measured by the Pfirrmann magnetic resonance imaging classification system (grade I–V) of lumbar DDD (Pfirrmann et al., 2001). Our hypothesis is that upregulation of MMP and ADAMTS expression implicated in disc ECM destruction is modulated by inflammation and upregulated under in chronic LBP. Considering therapeutic intervention and better diagnosing, we were interested to correlate such expression to other clinical parameters and biomarkers.

## 2 Materials and methods

### 2.1 Participants

We included 34 patients [9M, 25F] undergoing 1–2 level posterior lumbar instrumented spinal fusion L4/5 and/or L5/S1 with the placement of pedicle screws and an intervertebral cage in the disc space. All participants were radiologically evaluated with magnetic resonance imaging (MRI) and lumbar X-rays and clinical evaluation in the outpatient clinic by a senior spine surgeon. Inclusion criteria were: 18–65 of years of age; predominantly LBP with DDD and/or spondylolisthesis or predominantly radiculopathy with severe foraminal stenosis with or without spondylolisthesis; scheduled 1–2 level fusion with pedicle screws and placement of a transforaminal lumbar interbody cage; and pain (either predominantly radiculopathy or LBP with or without radiculopathy). Exclusion criteria were: symptom duration <6 months; previous spine surgery; rheumatoid arthritis; or ongoing steroid treatment. None of the participants had any significant somatic or psychiatric history on clinical examination other than those related to their lumbar degenerative disorder. The study was approved by the Danish ethics committee (H-17026301). All participants provided written informed consent according to the Declaration of Helsinki.

### 2.2 Informed consent statement

Informed consent was obtained from all subjects involved in the study.

### 2.3 Pfirrmann MRI grading system

The degree of disc degeneration was assessed for each patient using the 5-level Pfirrmann MRI grading system on T-2 weighted sagittal MRI sequences. A Pfirrmann grades I and II indicates a non-degenerative disc, whereas grade III, IV, and V signify an increasingly degenerative disc (Pfirrmann et al., 2001).

### 2.4 Tissue sampling

Following decompression and exposure of the IVD, the surgeon harvested the AF and the NP before preparation of the disc space for placement of the intervertebral cage. A window of the AF was cut open with a scalpel, and any adherent NP to the underside of the AF sample was cut off using a new scalpel. Next, several samples of NP were retrieved by gentle use of forceps in the disc space. The samples were immediately frozen on dry ice and stored at -80 ​°C until further processing. Only NP tissues were used for this study. During the whole sampling procedure, the surgeon carefully avoided contact of the sampling instruments with the surrounding tissue.

### 2.5 RNA extraction and cDNA synthesis

RNA extraction from NP was performed using TRIzol (Sigma Aldrich., Denmark), according to manufactures protocol, and the RNA content was quantified using a Nanodrop 2000 spectrophotometer (Thermo Scientific). Initially, the tissue was digested at 37 ​°C on a waving shaker set at 40rpm for 1 ​h in Dulbecco's Modified Eagle Medium containing 2 ​mg/ml proteinase (Pronase; Qiagen). Then, digestion was terminated by adding 10% fetal bovine serum when the tissue appeared nearly digested, it is washed twice with phosphate-buffered saline. Finally, cDNA was synthesized from 0.2 ​μg of RNA using ImProm-II^TM^ Reverse Transcription System (Promega, USA).

RT-qPCR was performed using a Light Cycler 480 Real-Time PCR System (Roche Diagnostics, IN) with SYBR Green I Master Mix for 40 cycles in a fixed sequence at 94 ​°C for 30 ​s, 60 ​°C for 15 ​s, and 72 ​°C for 15 ​s. Amplicons were generated using the primer sets listed in Table 1. We started performing the gene expression analysis using RT-qPCR by validating the housekeeping genes, so we initially analyzed and validated the expression of 5 housekeeping genes as previously described (Zhou et al., 2014) in the IVD using microsoft excel-based software programs, geNorm (ver. 3.5), NormFinder (Ver 0.953), and BestKeeper. From this analysis in our tissues, three genes listed in Table 1 were found to be stably expressed in the validation and accordingly used as housekeeping genes. Statistical algorithms were used to evaluate the stability of candidate reference genes, and then the overall ranking of the 5 candidate reference genes was determined according to the method described by Chen et al. (2011). Expression of all the MMPs and ADAMTSs were measured relative to the geometric mean of 3 housekeeping genes.

**Table:1:** List of primers used in this study.

Gene	Forward (5’- 3’)	Reverse (5’- 3’)
**TNF- α**	TTC CTG ATC GTG GCA GGC	GCT GAT TAG AGA GAG GTC CCT G
**IL-6**	TTC GCT CTT CCA GTT GGA CT	CAC CAG GGG AAG AAT CTG AG
**SDHA**	CGA GCT GCA TTT GGC CTT TC	TTG ATT CCT CCC TGT GCT GC
**LDHA**	GCC TGT ATG GAG TGG AAT GAA	CCA GGA TGT GTA GCC TTT GAG
**β-Actin**	TGG AAC GGT GAA GGT GAC AG	AAC AAC GCA TCT CAT ATT TGG AA
**Aggrecan**	GTG CCT ATC AGG ACA AGG TCT	GAT GCC TTT CAC CAC GAC TTC
**MMP1**	GCC ATC ACT TAC CTT GCA CT	AGA CAC CAC ACC CCA GAA CA
**MMP3**	TCC TAC TGT TGC TGT GCG TG	AGG TTC ATG CTG GTG TCC TC
**MMP2**	TAC AGG ATC ATT GGC TAC ACA CC	GGT CAC ATC GCT CCA GAC T
**MMP10**	TGA GTT TGA CCC CAA TGC CA	GTC TTC CCC CTA TCT CGC CT
**MMP13**	GGC TTA GAG GTG ACT GGC AA	ATC AGG AAC CCC GCA TCT TG
**ADAMTS1**	AGG ATG AAA CGC CGG AAC AA	CCC CAC CAC AAG ACA AGT GA
**ADAMTS4**	ACT GGT GGT GGC AGA TGA CA	TCA CTG TTA GCA GGT AGC GCT TT
**ADAMTS5**	TCG GGA GGA TTT ATG TGG GC	TGG AAT CGT CAT GGG AGA GG

### 2.6 Statistical analysis

Statistical analysis was performed with the Graphpad Prism 8.2 software. The correlation of mRNA levels and clinical data was assessed using Spearman's correlation coefficient. Similarly, a p-value of <0.05 is considered statistically significant for all comparisons.

## 3 Results

### 3.1 Expression of specific MMPs and ADAMTSs correlate strongly with Pfirrmann MRI grades

**Fig. 1:**
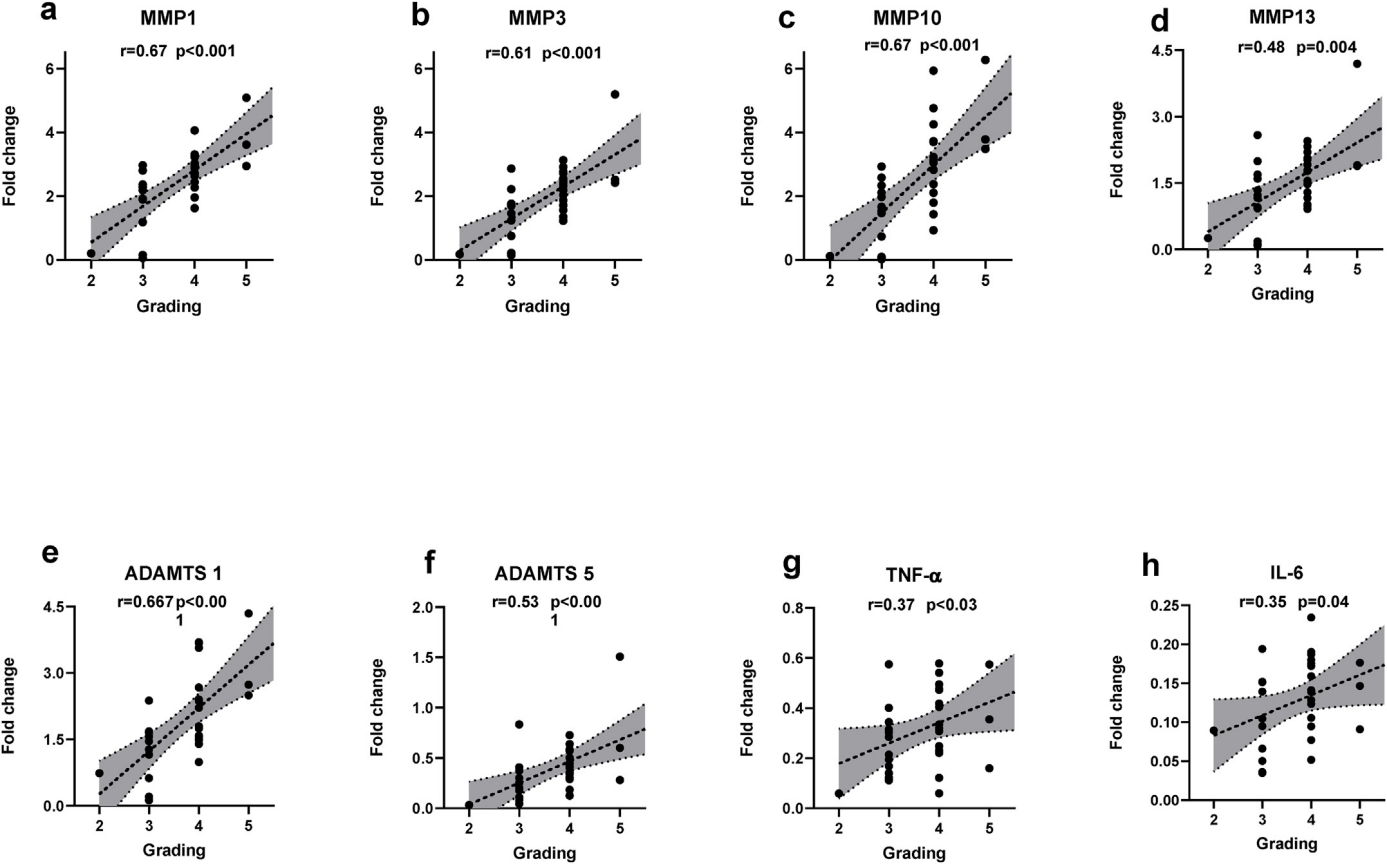
Expression of MMPs and ADAMTSs correlate with Pfirrmann MRI grades: Significant positive correlation observed between Pfirrmann MRI grades and expression of **(A)** MMP1 (ρ ​= ​0.67, p<0.0001), **(B)** MMP3 (ρ ​= ​0.61, p ​= ​0.0002), **(C)** MMP10 (ρ ​= ​0.671, p<0.0001), **(D)** MMP13 (ρ ​= ​0.48, p ​= ​0047), **(E)** ADAMTS1 (ρ ​= ​0.679, p<0.0001), **(F)** ADAMTS5 (ρ ​= ​0.53, p ​= ​0.001), **(G)**TNF-α (ρ ​= ​0.37, p<0.03) and (H) IL-6 (ρ ​= ​0.35, p ​= ​0.04) . p<0.05 (two tailed) are considered statistically significantly for all comparisons. ρ ​= ​spearmen’s correlation coefficient.

After surgical collection of the IVD specimens, mRNA expression analysis was performed using RT-qPCR on NP tissue. We found significant positive correlations between Pfirrmann grade and expression of MMP1 (r ​= ​0.67, p<0.0001) (Fig. 1A), MMP3 (r ​= ​0.61, p ​= ​0.0002) (Fig. 1B), MMP10 (r ​= ​0.671, p<0.0001) (Fig. 1C), MMP-13 (r ​= ​0.48, p ​= ​0047) (Fig. 1D), ADAMTS-1 (r ​= ​0.679, p<0.0001) (Fig. 1E), ADAMTS5 (r ​= ​0.53, p ​= ​0.001) (Fig. 1F). Performing a post-hoc analysis with the expression of pro-inflammatory cytokines, we also found a significant positive correlation with Pfirrmann grade and expression of TNF-α (r ​= ​0.37, p < 0.03) (Fig. 1G) and IL-6 (r ​= ​0.35, p ​= ​0.04) (Fig. 1H). We did not find any statistical correlation between Pfirrmann grade and expression of MMP2, ADAMTS4, IL-1β, NGF or BDNF (data not shown).

### 3.2 Correlation between mRNA expression of MMPs and ADAMTSs

**Fig. 2:**
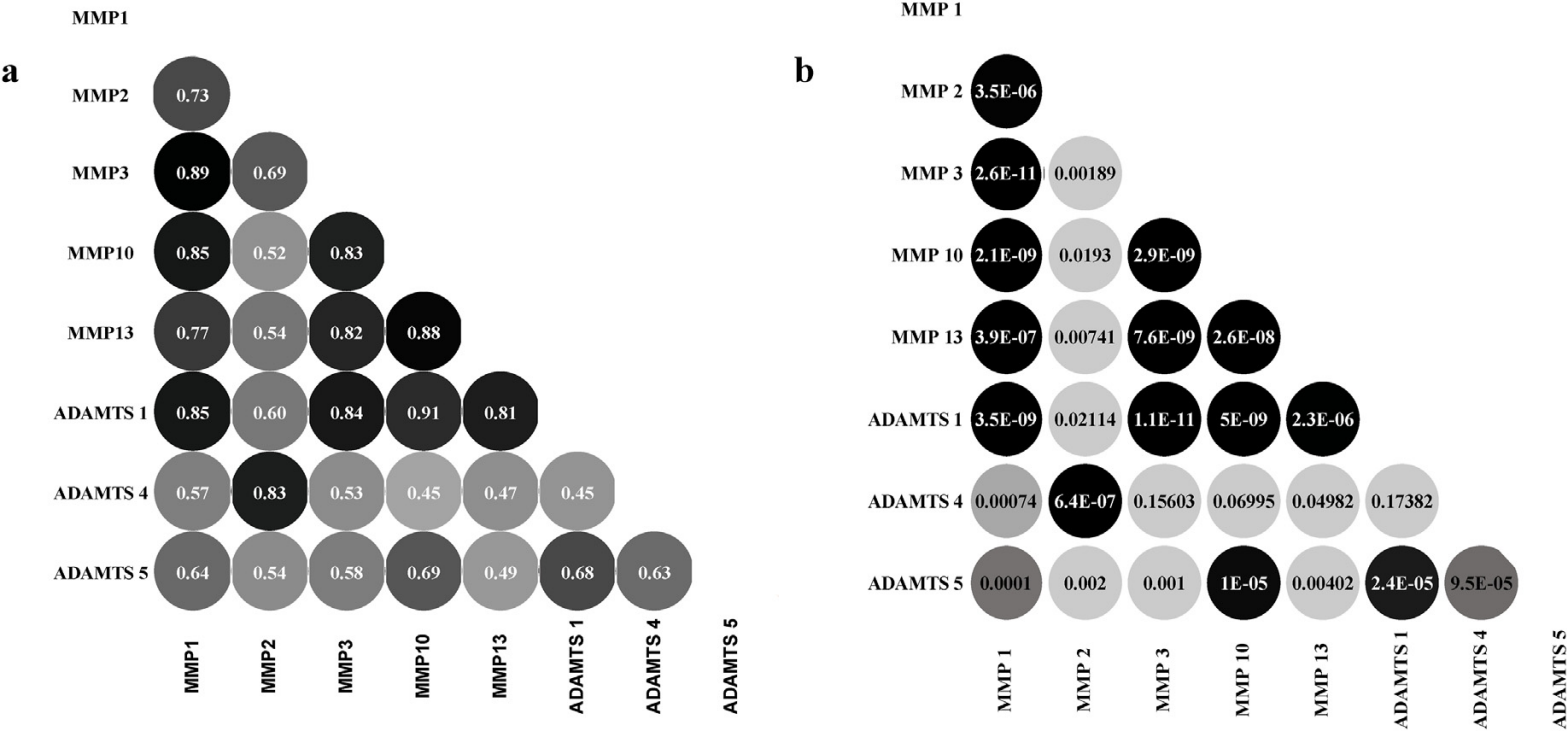
Correlation between mRNA expression of MMPs and ADAMTSs: Multiple significant positive correlations were found concerning the mRNA expression status of almost all of the MMPs and ADAMTS studied. **(A)** shows the correlations(ρ) between MMPS and ADAMTSs, and **(B)** shows corresponding significance values (p). p<0.05 (two-tailed) are considered statistically significant for all comparisons. ρ ​= ​spearmen’s correlation coefficient.

Since the transcripts are involved in proteolytic activity, we performed a post hoc pair-wise correlation analysis between expression of MMPs and ADAMTSs levels and demonstrated a strongly significant positive correlations between the expressions of these genes (Fig. 2A and B).

### 3.3 Correlation between transcript levels of aggrecan with age

**Fig. 3:**
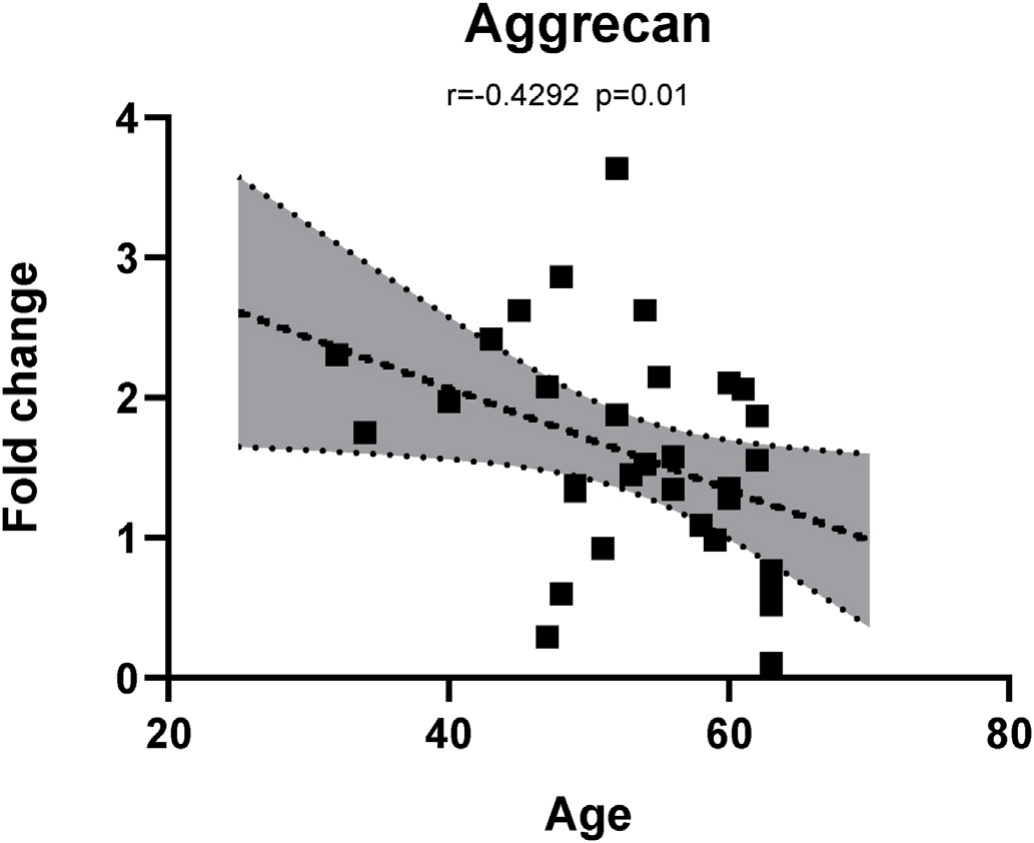
Correlation between expression of ECM gene aggrecan with age: Significant negative correlation observed between the expression of aggrecan with the age of the patients undergoing lumbar spine fusion (r ​= ​-0.42, ρ ​= ​0.012). p<0.05 (two-tailed) are considered statistically significant for all comparisons. ρ ​= ​spearmen’s correlation coefficient.

We observed a relation between expression level of aggrecan and the age of the patients (r ​= ​-0.42, p ​= ​0.012) (Fig. 3), Similarly, aggrecan showed significant positive correlations with expression of MMP (2, 10, 13), ADAMTS (1, 4, 5), but none with pro-inflammatory cytokines. Similarly, we observed negative correlations between expression of MMP2, ADAMTS4 and age of the patients (data not shown).

### 3.4 Correlation of transcript levels of MMPs and ADAMTSs with pro-inflammatory cytokines

**Fig. 4:**
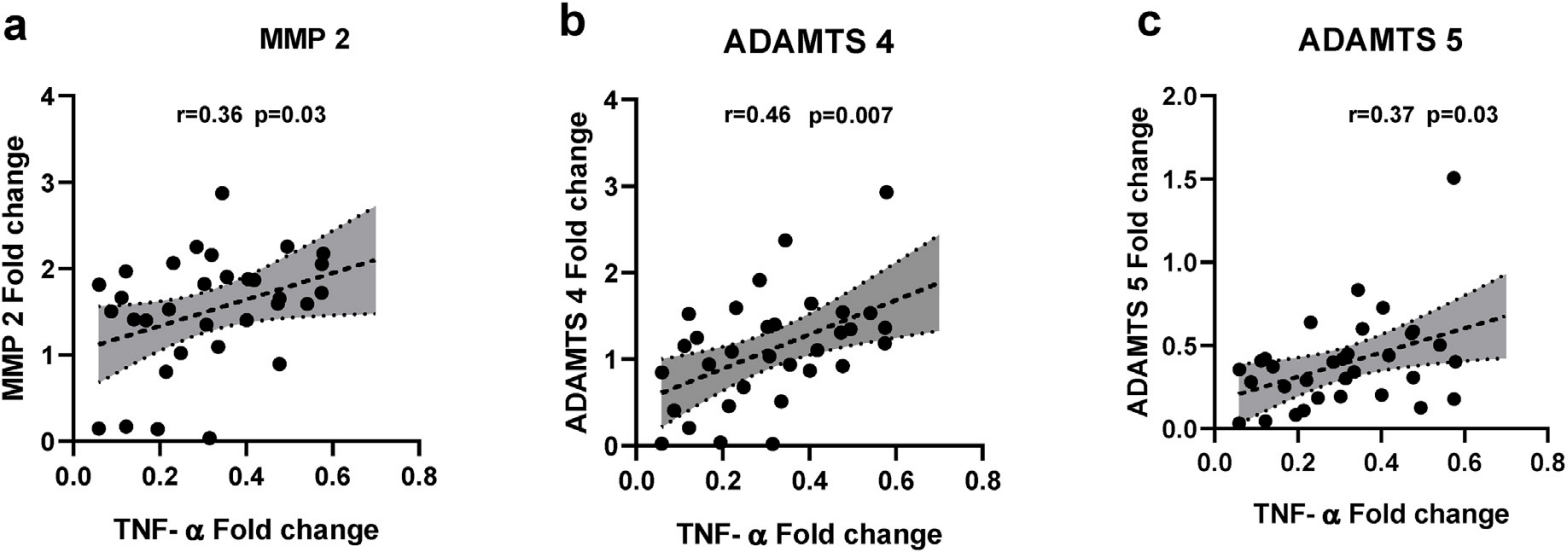
Correlation between pro-inflammatory cytokine TNF-α expression and MMP and ADAMTS***:*** A post hoc analysis of the intercorrelation between TNF-α mRNA expression to the expression of different MMPs and ADAMTSs revealed significant positive correlations between **(A)** TNF-α and MMP-2 (r ​= ​0.36, p ​= ​0.039), **(B)** ADAMTS4 (r ​= ​0.46, p ​= ​0.007), and **(C)** ADAMTS5 (r ​= ​0.33, p ​= ​0.03). p<0.05 (two-tailed) are considered statistically significant for all comparisons.

**Fig. 5:**
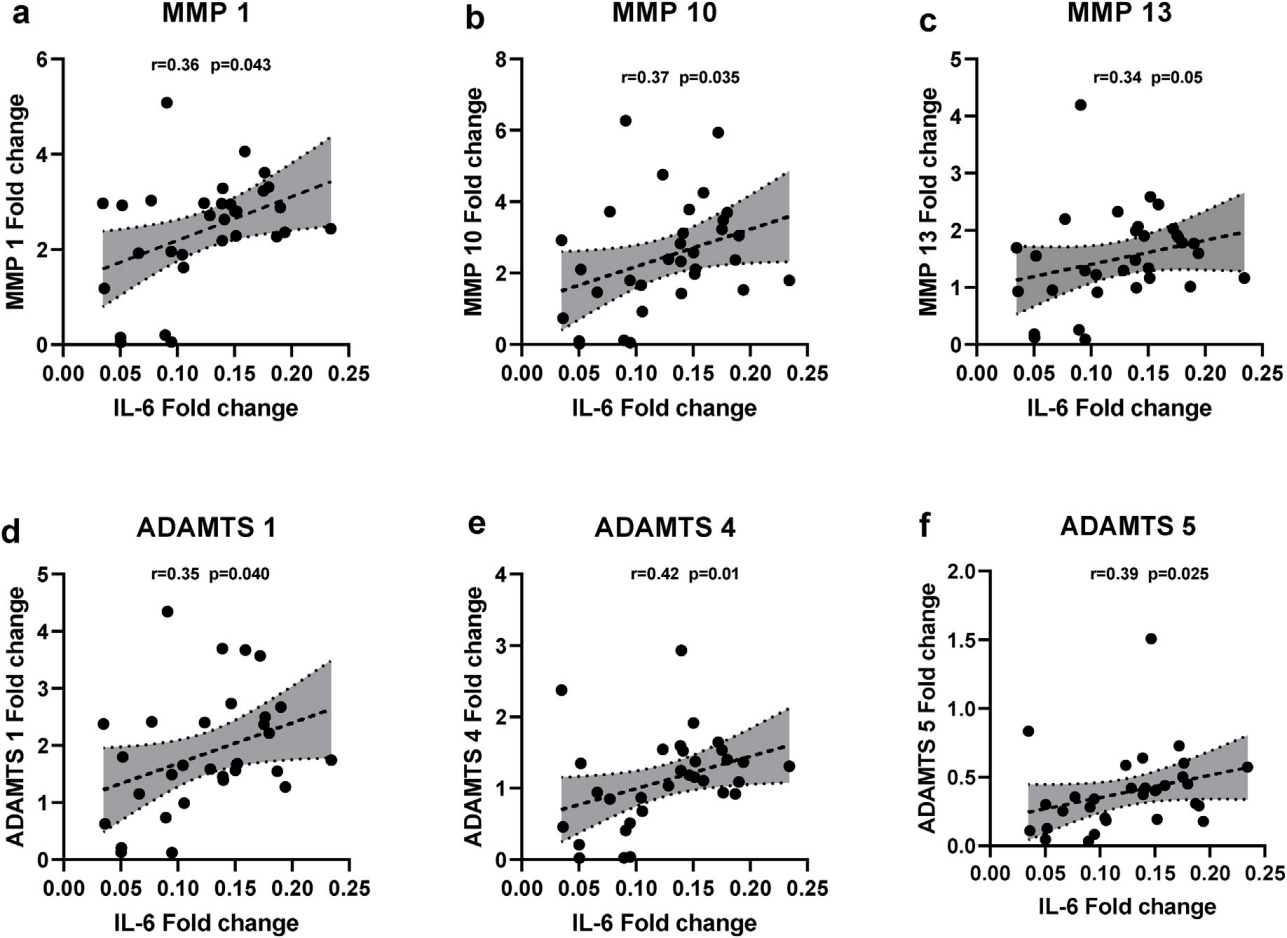
Correlation between pro-inflammatory cytokine IL-6 expression and MMP and ADAMTS: A post hoc analyses of the intercorrelation between IL-6 mRNA expression to the expression of different MMPs and ADAMTSs revealed significant positive correlations between **(A)** IL-6 and MMP1(r ​= ​0.36, p ​= ​0.04), **(B)** MMP10(r ​= ​0.36, p ​= ​0.037), **(C)** MMP13 (r ​= ​0.34, p ​= ​0.05), **(D)** ADAMTS1 (r ​= ​0.35, p ​= ​0.049), **(E)** ADAMTS4 (r ​= ​0.42, p ​= ​0.016), **(F)** ADAMTS5 (r ​= ​0.39, p ​= ​0.024). p<0.05 (two tailed) are considered statistically significantly for all comparisons.

To elucidate the mechanisms behind disc degeneration, we conducted a post hoc analyses of the intercorrelation between the individual cytokine mRNA’s expression to the expression of different MMPs and ADAMTSs. Interestingly, a relatively strong and significant positive correlations between TNF-α and MMP2 (r ​= ​0.36, p ​= ​0.039) (Fig. 4A), ADAMTS4 (r ​= ​0.46, p ​= ​0.007) (Fig. 4B), and ADAMTS5 (r ​= ​0.33, p ​= ​0.03) (Fig. 4C), but not to the other proteases. Similarly, we found significant positive correlations between IL-6 and MMP1 (r ​= ​0.36, p ​= ​0.04) (Fig. 5A), MMP10 (r ​= ​0.36, p ​= ​0.037) (Fig. 5B), MMP13 (r ​= ​0.34, p ​= ​0.05) (Fig. 5c), ADAMTS 1 (r ​= ​0.35, p ​= ​0.049) (Fig. 5D), ADAMTS4 (r ​= ​0.42, p ​= ​0.016) (Fig. 5E), ADAMTS5 (r ​= ​0.39, p ​= ​0.024) (Fig. 5F), but no correlation with MMP (2, 3, 13).

## 4 Discussion

In the present study, we have demonstrated significant positive correlations between Pfirrmann MRI grades of disc degeneration and the mRNA expression profile of MMP (1, 3, 10, 13) and ADAMTS (1, 5) in the NP biopsies from LBP patients undergoing lumbar spine fusion surgery. A likely explanation for for these results is a loss of collagen type II and aggrecan, which are essential components of the NP extracellular matrix and molecular targets for the majority of MMP's and ADAMTS's results in ECM breakdown and progression of disc degeneration. Even some proteases and their target molecules are affected under disc degeneration, the present study provides a comprehensive analysis of the expression of several genes, and the expression of these in the disc tissues and in correlation with several clinical markers.

### 4.1 Regulation of gene expression of MMPs and ADAMTSs with degenerative changes in IVD

Non-degenerated IVD expresses MMPs, although their expression is minimal (Weiler et al., 2002). Previous studies have shown increased expression of different MMPs in degenerated IVD tissues (Weiler et al., 2002; Le Maitre et al., 2004, 2006; Roberts et al., 2000; Bachmeier et al., 2009). These studies indicate that the ECM loss by proteolytic cleavage is key in degeneration, shifting the disc dynamics towards increased catabolism leading to a loss of function and pain generation. MMP-1 belongs to a subclass of collagenases that break down the fibrillar collagens type I, II and III dominantly present in NP and inner annulus fibrosus (Roberts et al., 1991). The present study showed positive correlation between MMP-1 and degeneration grade. These findings are supported by previous studies (Wang et al., 2015) which showed that with the degeneration an increase in the proportion of NP cells displaying MMP1-immunoreactivity increases. Our results on MMP-3 are also in agreement with previous histopathological observations reporting positive correlation between expression of MMP3 and MRI grading in degenerated disc (Canbay et al., 2013). MMP13 is more potent in cleaving collagen type II than MMP1 (Goupille et al., 1998). Previous studies demonstrated more MMP-13 immunoreactive cells within the NP of degenerated discs where collagen II is most abundant (Le Maitre et al., 2004; Roberts et al., 2000). Together with the present data a role of MMP13 in degeneration, probably via collagen II loss from the ECM is proposed, further suggesting that ECM loss due to collagen breakdown is driven by specific MMPs.

Several studies have shown ADAMTS in normal IVD, which signifies their role in normal tissue remolding and homeostasis (Le Maitre et al., 2004; Pockert et al., 2009). Our study showed a significant positive correlation between the NP expression of ADAMTS1 and ADAMTS5, but not ADAMTS4, to the Pfirrmann MRI grading system supporting a previous study conducted on human degenerated endplates. Here a marked upregulation of ADAMTS5 but not ADAMTS4 is reported, and it is concluded that ADAMTS5 and TNF-α play important role in degenerative endplates-induced low back pain (Chen et al., 2014). Similarly, work on NP cell cultures showed that expression of ADAMTS-5 increased under senescence induced by continuous passage of cultures (Le Maitre et al., 2007). Patel et al. (2007) found a higher level of ADAMTS4 in advanced level degeneration whereas the content of ADAMTS-5 protein levels does not change in contrast to our mRNA data.

Our post-hoc analysis on the pair-wise correlations showed co-expression of MMP10 with other MMPs and ADAMTSs. Along with the proteolytic activity of MMP10, it has been shown that MMP10 is a potent activator of MMP pro-enzymes (Barksby et al., 2006; Nakamura et al., 1998). These pair-wise correlations may explain that several MMP family members are activated simultaneously. Previous studies describe this activation of pro-MMPs by MMP10 as "super activation" (Barksby et al., 2006; Windsor et al., 1993). Such potent and convergent activation of these enzymes might dynamically shift the ECM metabolism to more catabolism by favoring MMP activity over inhibitors. Notably, there were few patients in the low and high grading groups (Grade 2 and 5), but these alone were not essential in the analysis. Even if these patients were excluded from the data analysis, we would have reached the same conclusion.

### 4.2 Expression of aggrecan in relation to age of the patients

Interestingly, we found a significant negative correlation between the expression of aggrecan and age, and this is independent of the disc degradation, Aged disc mainly contains aggrecan in a non-aggregated form and decreased glycosaminoglycan chain length which might be derived from proteolytic damage (Roughley, 2004). This non-aggregated aggrecan might not have the same functional ability as intact aggregates because their sizes and matrix interactions are diminished (Buckwalter, 1995; Adams et al., 1977). Hence age-related ECM molecular alterations cause structural integrity and biomechanical function loss is independent of the degeneration (Vo et al., 2016).

### 4.3 Regulation of inflammatory pathways on the expression of MMPs and ADAMTSs

Cytokines also up-regulate a wide variety of catabolic mediators like ADAMTS4, ADAMTS5, MMP1, MMP2, MMP13, MMP14, which suppress the expression of essential ECM genes (Le Maitre et al., 2005; Séguin et al., 2005; Wang et al., 2011; Tian et al., 2013). The link between cytokine expression and MMPs could be important because Séguin et al. (2008) showed that TNF-α induced MMP2 activity by controlling MMP14 expression occurs mainly through the extra cellular signal-regulated kinase pathway. Similarly, we also found significant positive correlations between the expression of TNF-α and ADAMTS4/ADAMTS5, which is in line with a report demonstrating that TNF-α and IL-1β regulate expression of the same ADAMTS subtypes through mitogen-activated protein kinase and nuclear factor kappa light chain enhancer of activated B cells signalling pathways (Séguin et al., 2006, 2008). Furthermore, IL-6 also synergistically potentiates the catabolic actions of IL-1β and TNF-α (Studer et al., 2011), and when treated with a cocktail of cytokines, a significant decrease in proteoglycan synthesis and an increase in MMP13 occur (Studer et al., 2011). Together, our findings suggest the critical role of cytokines in ECM metabolism and enable us to propose the role of intracellular pathways like mitogen-activated protein kinase and nuclear factor kappa light chain enhancer of activated B cells signalling pathways in regulating the expression of these proteases, and therapeutic blockage of one of these would be expected to prevent loss of ECM. Similarly, it would be interesting to study these associations with Modic type changes because a recent study by Chen et al. (2014) showed that disc degeneration is one factor leading to different types of Modic changes and suggested severe degeneration means severe endplate damage and hence type II changes often occur. However, it is now intriguing to implicate therapeutic or preventive strategies from our study, even further studies are obviously needed to confirm their therapeutic potential.

## 5 Conclusion

Our results showed an imbalance between catabolism and anabolism of IVD matrix components. We showed that most MMPs and ADAMTSs are expressed in NP, and their expression levels increase with degeneration grade, suggesting the role of these proteases in ECM breakdown and progression of degeneration. Our results also propose that stimuli coming from the release of inflammatory cytokines participate in the regulation of MMPs and ADAMTSs, and dysregulation of their activity can increase the disc's structural loss. The study present interesting new observations, due to a full inclusion and exclusion criteria for patient recruitment in the study design, and the inclusion of expression data for many proteases simultaneously. Together with the clinical data and the MRI data, we see very strong correlations between the biomarkers and the clinical observations. Our results contribute to understanding the role of different MMPs and other aggrecanases in disc degeneration etiology with the potential to integrate novel biomarkers in diagnosis, therapy effects, and the prognosis for patients with DDD.

## Disclosure

The authors have declared no conflict of interest.

## Funding disclosure

This study is funded by the 10.13039/100008368Gigtforeningen and the 10.13039/501100008340Elsass Foundation.

## Author's contribution

JDM, RBA, and LMJ performed study design, and SSA conducted experiments. SSA and JDM did the data analysis, contributed both to data interpretation; SSA wrote the first draft manuscript, and all authors approved the final manuscript.

## Declaration of competing interest

The authors declare that they have no known competing financial interests or personal relationships that could have appeared to influence the work reported in this paper.
